# Multi-Platform-Based Analysis Characterizes Molecular Alterations of the Nucleus in Human Colorectal Cancer

**DOI:** 10.3389/fcell.2022.796703

**Published:** 2022-02-21

**Authors:** Wei Zhang, Minmin Wu, Xucan Gao, Chiyu Ma, Huixuan Xu, Liewen Lin, Jingquan He, Wanxia Cai, Yafang Zhong, Donge Tang, Min Tang, Yong Dai

**Affiliations:** ^1^ Department of Clinical Medical Research Center, The Second Clinical Medical College, Jinan University (Shenzhen People’s Hospital), Shenzhen, China; ^2^ South China Hospital, Health Science Center, Shenzhen University, Shenzhen, China; ^3^ Key Laboratory of Clinical Laboratory Diagnostics of Ministry of Education, Chongqing Medical University, Chongqing, China

**Keywords:** nucleus, colorectal cancer, structural variations, immune microenvironment, multiomics

## Abstract

**Background:** The disturbed molecular alterations of nucleus may promote the development of colorectal cancer (CRC). A multi-platform-based analysis of nucleus of CRC patients helps us to better understand the underlying mechanism of CRC and screen out the potential drug targets for clinical treatment. However, such studies on nucleus in human CRC are still lacking.

**Methods:** We collected the cancerous and para-cancerous tissues from eight CRC patients and performed a multiplex analysis of the molecular changes of the nucleus, including structural variations (SVs), DNA methylation, chromatin accessibility, proteome and phosphorproteome.

**Results:** In our study, we revealed a significant molecular change of nucleus of CRC patients using our original proteomic and phosphorylomic datasets. Subsequently, we characterized the molecular alterations of nucleus of CRC patients at multiple dimensionalities, including DNA, mRNA, protein and epigenetic modification. Next, we found that the great molecular changes of nucleus might affect the biological processes named endocytosis and ubiquitin-mediated proteolysis. Besides, we identified DYNC1LI2 and TPR as the potentially hub proteins within the network of nuclear genes in CRC cells. Furthermore, we identified 1905 CRC-specific SVs, and proclaimed 17 CRC-specific SVs were probably associated with the disturbance of immune microenvironment of CRC patients. We also revealed that the SVs of CXCL5, CXCL10 and CXCL11 might be the core SVs among all the immune-relevant SVs. Finally, we identified seven genes as the upstream transcriptional factors potentially regulating the expression of nuclear genes, such as YY1 and JUN, using a multi-omics approach.

**Conclusion:** Here, we characterized the molecular changes of nucleus of CRC patients, disclosed the potentially core nuclear genes within the network, and identified the probable upstream regulator of nucleus. The findings of this study are helpful to understand the pathogenic molecular changes of nucleus in CRC patients and provide a functional context for drug development in future.

## Introduction

Colorectal cancer (CRC) is one of the most common tumors in the world, ranking the third among all malignant tumors (Bray et al., 2018). With the improvements of surgery and novel targeted and immune drugs, the 5-year survival rate of CRC patients has been increased in recent years (Siegel et al., 2020). However, there still are 149,500 new cases and 52,980 deaths caused by CRC in the United States a year in 2020 (Siegel et al., 2020). Therefore, it is of great significance to understand the molecular changes and functional genes of human CRC.

Frequently, tumors may be induced by the changes of several genes at multiple molecular levels. Nowadays, omics studies are emerging as an effective way to study tumorigenesis, which one-time access to most intracellular information in cells or tissues (Rusk, 2019), such as genomic and epigenomic studies. At present, mono-omics techniques have already been widely used in detection of molecular defects of tumors, but it is not sufficient to elucidate the complex mechanism of carcinogenesis (Rusk, 2019). Therefore, we need the method of multi-omics cross-analysis to study the same patients at distinct molecular levels to understand the functional context of tumors. In the previous studies, we performed a multi-omics investigation of cancerous and para-cancerous tissues of CRC patients, revealing the dysfunctions of mitochondria in CRC patients [(Zhang et al., 2021a; Zhang et al., 2021b)].

It is well known that genetic mutations, chromatin instability and some other “nuclear anomalous events” play core roles in the initiation and progression of CRC (Choong and Tsafnat, 2012; Okugawa et al., 2015). Until now, our knowledge towards the nucleus is more in the view of genome, but the changes of nucleus at other molecular dimensionalities are barely known. In this study, we disclosed the global molecular changes of nucleus in CRC patients in the view of structural variations (SVs), DNA methylation, chromatin accessibility, and proteome and phosphoproteome using our original datasets. Besides, we analyzed the network of the nuclear genes in CRC patients and searched for the hub genes within the network. Furthermore, we identified the probable upstream transcription factors (TFs) that regulated the activity of nucleus. Finally, we analyzed the global chromatin SVs of CRC patients and uncovered the probable pathogenic SVs.

## Materials and Methods

### Patients

The primary CRC samples were acquired from Shenzhen People’s Hospital, and this project was approved by the Ethics Committee of Shenzhen People’s Hospital (LL-KY-2019213). All the participants were voluntary and signed the informed consent forms. Eight patients with colorectal adenocarcinoma were eligible for inclusion in this study who had undergone surgical resection without prior radiotherapy or chemotherapy. Also, the hereditary colon cancer was excluded. The clinical information of CRC patients were described in Supplementary Table S1.

### Protein Extraction and Digestion

Sample collection was performed as described in our previous articles (Sato et al., 2011; Zhang et al., 2021a; Zhang et al., 2021b). Both tumor and normal adjacent tissues were obtained from the colon segment, and normal adjacent colorectal mucosa was collected from 5 cm away from the tumors. Then, the samples were stored in liquid nitrogen for at least 3 hours. Next, the frozen samples were ground to powder and uniformly mixed at 1:4 by volume with the lysis buffer containing 8 M urea (Sigma) and 1% protease inhibitor cocktail (156535140, Millipore). The resulting mixture was ultrasonic treated for three times on ice with a high-intensity ultrasonic processor (Scientology, China) and then centrifuged at 12,000 × *g* for 10 min at 4 °C to remove the fragments. Finally, the BCA kit (P0011-1, Beyotime) was used to determine the protein concentration of the supernatant. The protein-containing samples were incubated with 5 mM dithiothreitol (Sigma) at 56°C for 30 min, then mixed with 11 mM iodoacetamide (Sigma) and incubated at 37°C for 15 min in dark. Next, 100 mM triethylammonium bicarbonate (Sigma) were added to dilute the urea concentration to 2 M. Finally, the mixture was digested with trypsin (V9012, Promega) in two rounds. That is, the ratio of mixture and trypsin was 1:50 and 1:100, digested for12 h and 4 h respectively.

### LC-MS/MS Analyses

LC-MS/MS was performed as described in previous study (Sun et al., 2019). The trypsin digested polypeptide samples were dissolved in liquid chromatographic mobile phase A (0.1% formic acid solution, Fluka) and separated using an ultra-high phase system. After separation, the mixture was injected into NSI ion source for ionization and then analyzed by timsTOF Pro mass spectrometer (Bruker Daltonics, MA, United States). The second-order mass spectrometry scanning range was 100–1700m/z, and the fragmentation of precursors with charge states 0 to five was performed. Dynamic exclusion was set to 30 s in secondary MS. Secondary MS data were retrieved using Maxquant (V1.6.6.0) as Homo sapiens 9606 SP 20191115 (sequence 20,380).

### Whole Genome Bisulfite Sequencing (WGBS)

The genomic DNA was extracted with SMRTbell Express Template Prep Kit 2.0 (100–938-900, Pacific Bioscience) and tested for quality and concentration. DNA samples were segmayed by ultrasound for bisulfite transformation, and the DNA methylation sequencing library construction Kit EZDNA methylation-Gold ™ Kit (D5005, ZYMO Research) was used to connect single strand DNA fragments for PCR amplification. The amplified products were purified and tested for their integrity using a 2,100 Bioanalyzer (Agilent). Finally, the HiSeq X10 sequencing platform (Illumina) was used for the double-terminal sequencing of the library, and the results were compared with each methylation regions.

### Long-Read Whole Genome Sequencing

The high quality genomic DNA was obtained using the same method as WGBS. The DNA fragments with a growth of about 20 kb were fragmented using the g-GUBETM (520,079, Covaris) and enriched by the magnetic beads (100-317-100, Pacific Bioscience). The DNA fragments were added with stem rings to synthesize the sequences using DNA polymerase (101-731-100, Pacific Bioscience). The DNA quality was assessed and sequenced using the HiSeq X10 sequencing platform (Illumina), which produced paired 150 bp readings of the terminal.

#### Assay to detect transposase-accessible chromatin based on high throughput sequencing (ATAC-Seq)

The ATAC-seq was performed as reported in previous study (Buenrostro et al., 2015). In brief, the suspension conteining 50,000 single-cell was processed for transposition and purification. After the cell lysis, 2.5 μL Tn5 transpoase and 1× TD buffer were added to the remaining cell precipitate, 50 μL in total, and incubated at 37°C for 30 min. The DNA sample was then processed using the MinElute reaction Clearance Kit (51,306, QIAGEN), and the DNA libraries were generated using the TruePrep DNA Library preparation Kit V2 (TD501/TD502/TD503, Vazyme Biotecho) based on the Illumina sequencing platform. Subsequently, the StepOnePlus real-time PCR system was used to verify the quality of the library.The high-quality libraries were then sequenced using the HiSeq X Ten sequencing platform (Illumina) based on 150 bp paired-end reading.

### Databases and Software

The RNA-Seq datasets were acquired from The Cancer Genome Atlas (TCGA), including 51 healthy people and 647 CRC patients (colonic or rectal intestinal mucosa were examined using the RNA-Sequencing). The patients with incomplete survival time were excluded, and 538 patients were ultimately included in this study. The resulting expression matrix of the TCGA data sets has been uploaded as the Supplementary Information. The datasets of immune cell infiltration were obtained from Xcell database. Gene Ontology (GO) and Kyoto Encyclopedia of Genes and Genomes (KEGG) analysis were performed through DAVID database (https://david.ncifcrf.gov/). The subcellular enrichment was performed using WoLFPSOR v.0.2 software. The protein-protein interaction (PPI) network was constructed using Cytoscape and Metascape.

### Data Available

The datasets of proteome and phosphoproteome have been uploaded to the ProteomeXchange Consortium with the accessing number PXD021314 and PXD021318. The datasets of ATAC-seq, WGBS and LWGS have been stored in Sequence Read Archive (SRA) with the accessing number PRJNA693028.

### Statistical Analyses

*t*-test was utilized to calculate the significance of differentially expressed genes. Fisher’s exact test was used to analyze the significance of enrichment. *p* value <0.05 was considered statistically significant.

## Results

Integrated proteome and phosphoproteome revealed a tremendous molecular alteration of the nucleus of CRC patients.

**FIGURE 1:**
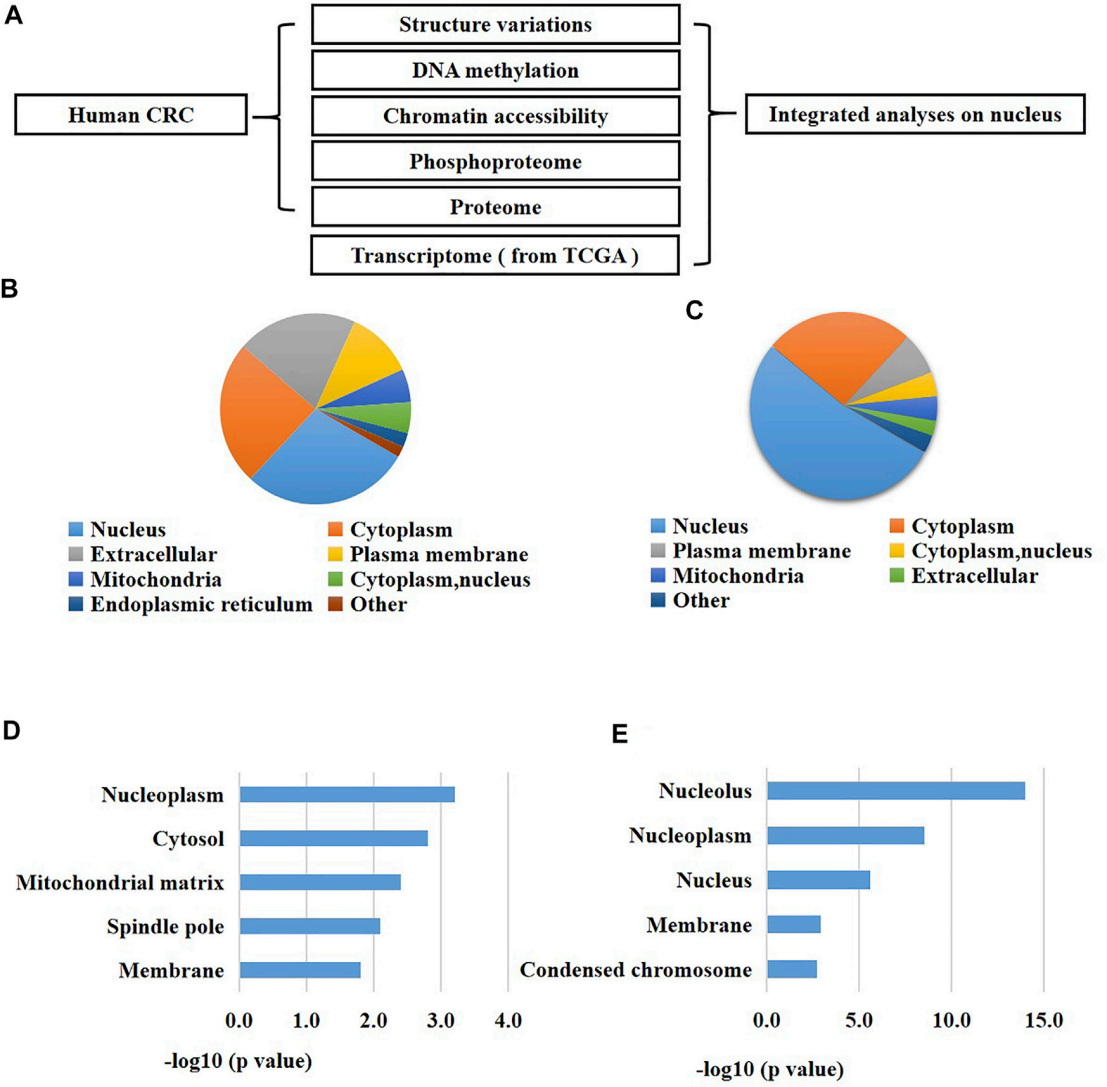
An integrated analysis revealed a significant change of nucleus in CRC patients. **(A)** A schematic overview of the study. **(B)** Subcellular enrichment analysis of the up-regulated proteins of CRC cells versus normal cells. **(C)** Subcellular enrichment analysis of all differentially expressed phosphorylation of CRC cells versus normal cells. **(D)** GO enrichment analysis of the up-regulated proteins of CRC cells versus normal cells. **(E)** GO enrichment analysis of all differentially expressed phosphorylation of CRC cells versus normal cells.

To uncover the molecular changes of nucleus of CRC cells, we first collected the cancerous tissues and the normal adjacent tissues from CRC patients (n = 6–8). Samples of two patients at each stage were pooled into a single one for the following test (Supplementary Table S1). We then performed a multiplex analysis of the CRC cells and normal cells using the data sets of our own, comparing the global SVs, DNA methylation, chromatin accessibility, proteome and phosphoproteome between these two groups (Figure 1A).

Since proteins are the fundamental units for cellular biological functions, we primarily analyzed the proteome and phosphoproteome of the CRC cells compared to the normal adjacent cells. As a result, we found 496 proteins were up-regulated and 597 proteins were down-regulated in CRC cells versus the normal cells using the proteomic analysis (fold changes >1.5, *p* < 0.05, Supplementary Table S1). Meanwhile, we identified 587 differentially expressed phosphorylation in CRC cells versus the normal cells using the phosphoproteomic analysis (fold changes >1.5, *p* < 0.05, Supplementary Table S1). Next, we performed enrichment investigations of the above differentially expressed proteins and phosphorylation using GO analysis and subcellular analysis. The results exhibited that many nuclear proteins were enriched in the expression-increased proteins (29% of all the up-regulated proteins) (Figure 1B). Similarly, a great number of nuclear proteins were also enriched in the altered phosphorylation (53% of all the differentially expressed phosphorylation) (Figure 1C). On the other hand, GO analysis also confirmed these results. Many expression-elevated proteins located in nucleoplasm and spindle pole (Figure 1D), and many differentially-expressed phosphorylation located in nucleolus, nucleoplasm, nucleus, membrane, and condensed chromosome. These results indicated that the nucleus were greatly changed during the initiation and development of human CRC. In addition, the abnormal alterations of phosphorylation of nuclear proteins might be closely related to the carcinogenesis of CRC.

The proteogenomic characterization of the nucleus of CRC patients.

**FIGURE 2:**
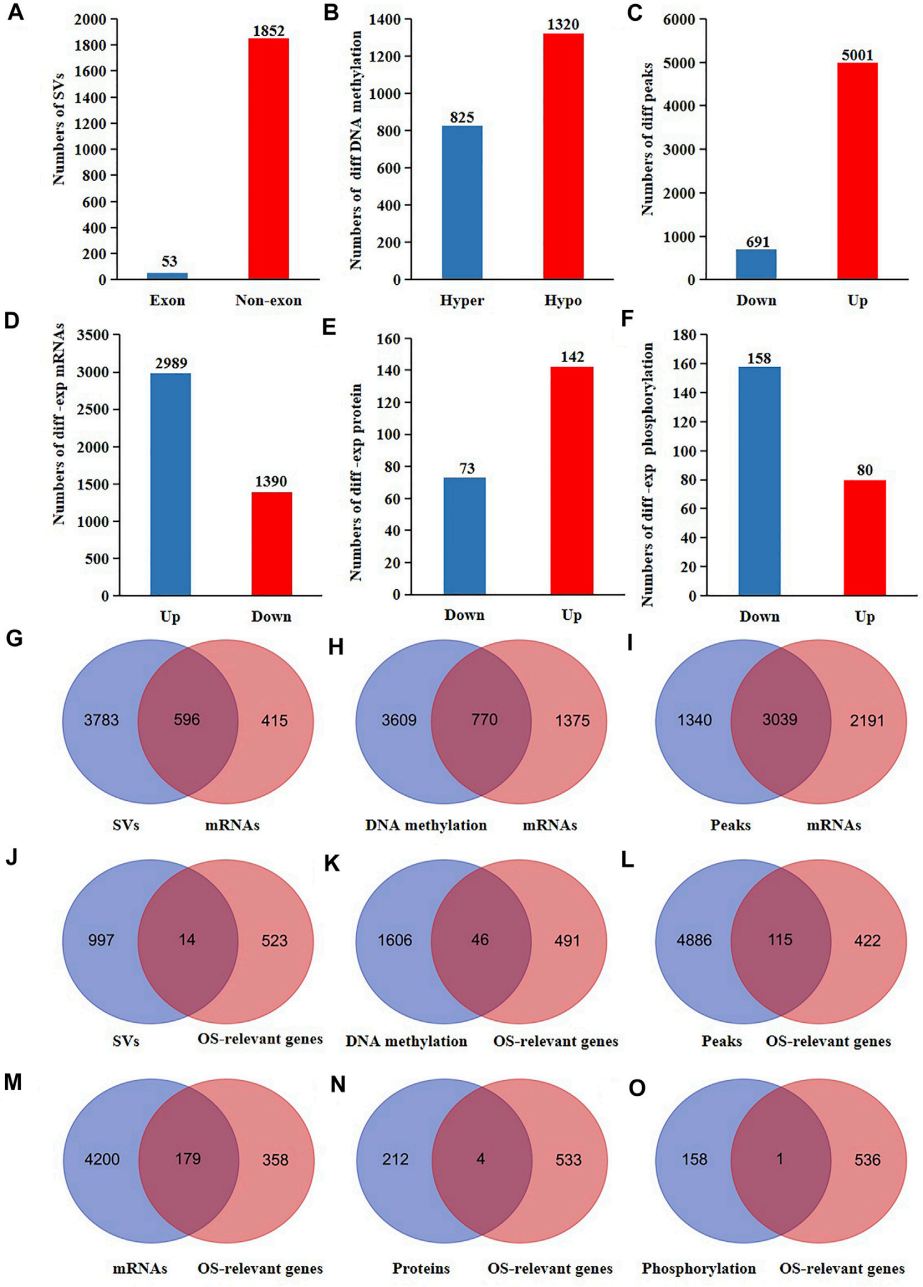
The proteogenomic alterations of nucleus of CRC patients. The different **(A)** SVs **(B)** DNA methylation **(C)** accessible chromatin peaks **(D)** mRNAs **(E)** proteins, and **(F)** phosphorylation of nuclear genes of CRC cells compared to normal cells. Venn analyses of the different **(G)** SVs **(H)** DNA methylation **(I)** accessible chromatin peaks and the differentially expressed mRNAs of nuclear genes of CRC cells compared to normal cells. Venn analyses of the different **(J)** SVs, **(K)** DNA methylation, **(L)** accessible chromatin peaks, **(M)** mRNA, **(N)** proteins, **(O)** phosphorylations, and the OS-relevant nuclear genes of CRC patients.

A comprehensive study of the molecular changes of the nucleus of CRC cells at distinct levels is helpful to enhance our understanding of the dysfunctional contributions of nucleus in carcinogenesis. Therefore, we investigated the SVs (n = 6), DNA methylation (n = 6), chromatin accessibility (n = 6), proteins (n = 8), and phospholation (n = 8) of all the nuclear genes (proteins having a nucleus location) in CRC cells versus normal cells. Moreover, we also analyzed the mRNA expression of all the nuclear genes using the RNA-seq datasets of 538 CRC patients and 51 healthy individuals downloaded from the TCGA. The results showed that 1852 nuclear genes had CRC-specific SVs at non-exon sites and 53 nuclear genes had CRC-specific SVs at exon sites (Figure 2A). 825 nuclear genes were hyper-methylated and 1320 nuclear genes were hypo-methylated in CRC cells compared to normal cells (Figure 2B). 5,001 nuclear genes had increased chromatin accessibility and 691 nuclear genes had decreased chromatin accessibility in CRC cells compared to normal cells (Figure 2C). The mRNA expression of 2,989 nuclear genes were up-regulated and the mRNA expression of 1390 nuclear genes were down-regulated in CRC cells compared to normal cells (Figure 2D). The protein expression of 142 nuclear genes were up-regulated and the protein expression of 73 nuclear genes were down-regulated in CRC cells compared to normal cells (Figure 2E). 80 nucleoproteins had more phosphorylation and 158 nucleoproteins had less phosphorylation in CRC cells compared to normal cells (Figure 2F).

It is well known that the genes transcription are influenced by SVs, DNA methylation, and chromatin accessibility. Therefore, we further explored the probable reasons for the altered mRNA expression of the nuclear genes in CRC cells. As a result, 596 expression-changed nuclear genes had SVs (60% of all expressed-changed nuclear genes) (Figure 2G). Among 2,145 nuclear genes with altered DNA methylation, the mRNA expression of 770 genes were altered in CRC cells (35.9% of all expressed-changed nuclear genes) (Figure 2H). Among 5,230 nuclear genes with altered chromatin accessibility, the mRNA expression of 3,039 genes were altered in CRC cells (58.1% of all expressed-changed nuclear genes) (Figure 2I). These results suggested that SVs might have a bigger influence on the transcription of the nuclear genes in CRC patients.

Proteogenomic changes of functional genes may affect tumor progression. Consequently, we searched for the molecular changes of the survival-relevant nuclear genes in CRC patients using the data sets from TCGA. We primarily performed a survival analysis of all mRNAs detected in colonic cells. The results showed that 537 genes were associated with the overall survival rates (OS) of CRC patients (Supplementary Table S1). Subsequently, we overlapped the 537 OS-relevant genes with all the different SVs, DNA methylation, chromatin accessibility, mRNAs, proteins, and phosphorylation of CRC cells. As a result, we found that 14 OS-relevant nuclear genes had CRC-specific SVs, 46 OS-relevant nuclear genes had hyper or hypo DNA methylation, 115 OS-relevant nuclear genes had different chromatin accessibility, 179 nuclear OS-relevant genes had differentially expressed mRNAs, four OS-relevant nuclear genes had differentially expressed proteins, and one OS-relevant nuclear genes had altered phosphorylation (Figures 2J–O). These results showed that both the genomic and post-translational modifications of nuclear genes were potentially related to the outcomes of CRC patients.

The phosphoproteomic analysis uncovered significant changes of phosphorylation of nuclear proteins of CRC patients and identified DYNC1LI2 and TPR as the potential hub genes within the nucleus network.

**FIGURE 3:**
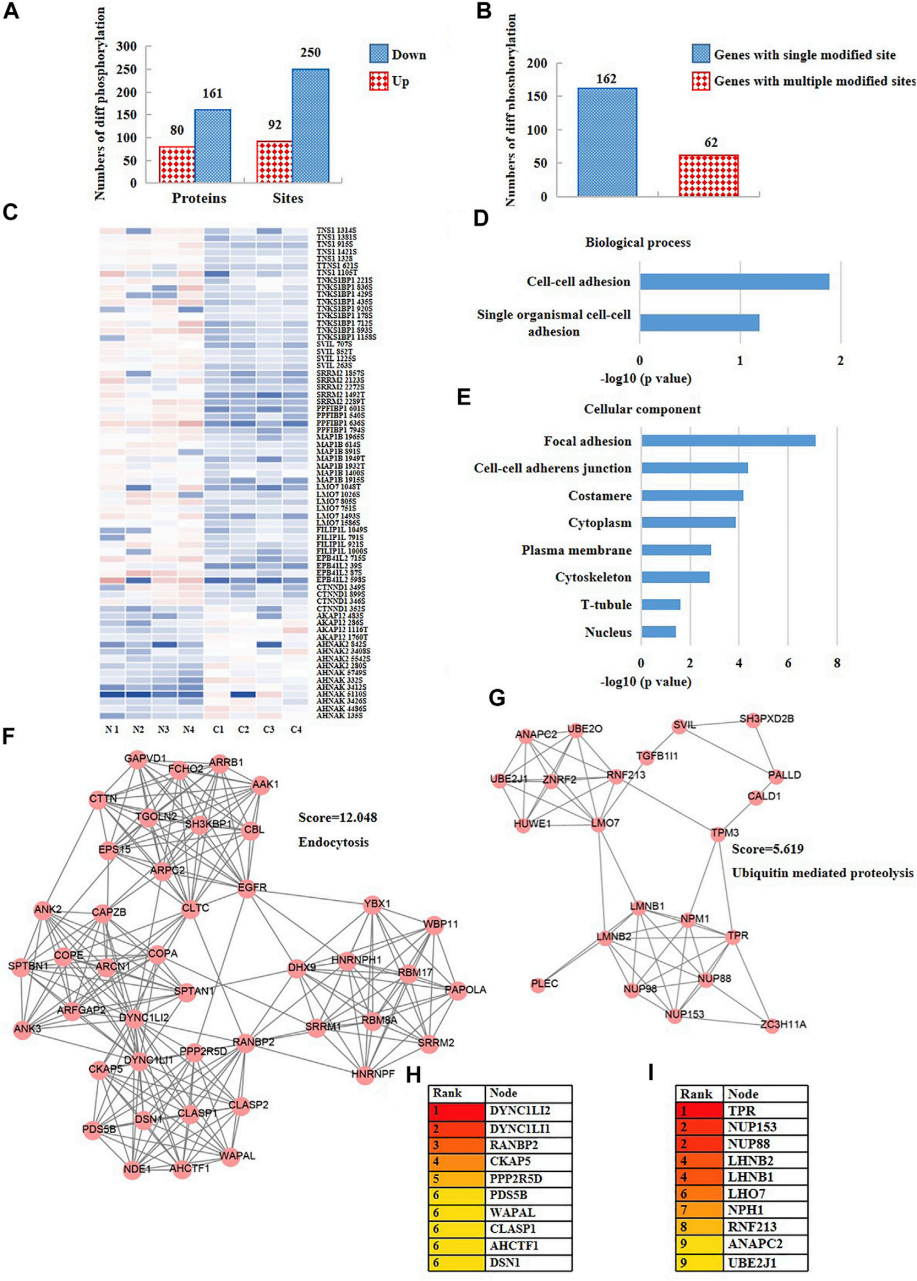
The phosphoproteomic analysis of nuclear proteins disclosed DYNC1LI2 and TPR potentially as the hub genes in CRC. **(A)** The numbers of differentially expressed phosphorylation of CRC cells versus normal cells. **(B)** The numbers of differentially expressed phosphorylation having one-site changes or having more than two sites of changes. **(C)** The heat map showing the phosphorylation content of nucleoproteins of CRC cells versus normal cells. **(D)** Biological process, and **(E)** cellular component enrichment analysis of all differentially expressed phosphorylation of CRC cells versus normal cells. **(F,G)** Two closely-tied groups within the network of all differentially phosphorylated nuclear proteins. **(H,I)** Hub proteins within the two closely-tied groups.

It is well known that phosphorylation is a type of post-translational modification that switches protein activity and regulates intracellular signal transmission. Altered phosphorylation contents in cells may cause tumors. According to the above results, the phosphoproteome of nuclear in CRC patients was significantly changed. To explore the cellular functions of the altered phosphorylation, we first calculated the numbers and sites of the different phosphorylation of nuclear genes of CRC cells versus the normal cells. As a result, the phosphorylation of 80 nuclear proteins were up-regulated and 161 proteins were down-regulated in CRC cells versus the normal cells. 92 phosphorylation sites were up-regulated and 250 sites were down-regulated in CRC cells versus the normal cells (Figure 3A, Supplementary Table S1). Among all the proteins with differentially expressed phosphorylation, 162 proteins only had one single differentially expressed phosphorylation site, and 62 proteins had two or more than two differentially expressed phosphorylation sites (Figure 3B, Supplementary Table S1). Subsequently, to understand the functions of the proteins with more than two differentially expressed phosphorylation, we analyzed the phosphorylation expression of 13 highly phosphorylated (n ≥ 4) nuclear proteins. The results showed that most of these phosphorylation were reduced in CRC cells versus the normal cells (Figure 3C). The KEGG and GO analysis revealed that the functions of these proteins were mainly about cell-cell adhesion and cytoskeletal proteins (Figures 3D,E, Supplementary Table S1). Furthermore, we constructed the network of the nuclear proteins with differentially expressed phosphorylation using Cytoscape and analyzed the closely connected protein groups using MCODE. As a result, the groups with the highest scores were relevant to the processes including endocytosis (score = 12.048) (Figure 3F) and ubiquitin-mediated proteolysis (score = 5.619) (Figure 3G). In addition, we analyzed the hub proteins within the two groups using Cytohubba. The results revealed that DYNC1LI2 (Figure 3H) and TPR (Figure 3I) were probably the most important genes within the network. Next, we investigated the phosphorylation of DYNC1LI2 and TPR in CRC cells compared to the normal cells using our original phosphoproteomic datasets. The results revealed that DYNC1LI2 (Figure 3H) and TPR (Figure 3I) were probably the most important genes within the network. Next, we investigated the phosphorylation of DYNC1LI2 and TPR in CRC cells compared to the normal cells using our original phosphoproteomic datasets. As a result, the phosphorylation of DYNC1LI2 at Ser196 was up-regulated and TPR at Ser2155 was down-regulated in CRC cells versus normal cells (Supplementary Figure S1).

**FIGURE 4:**
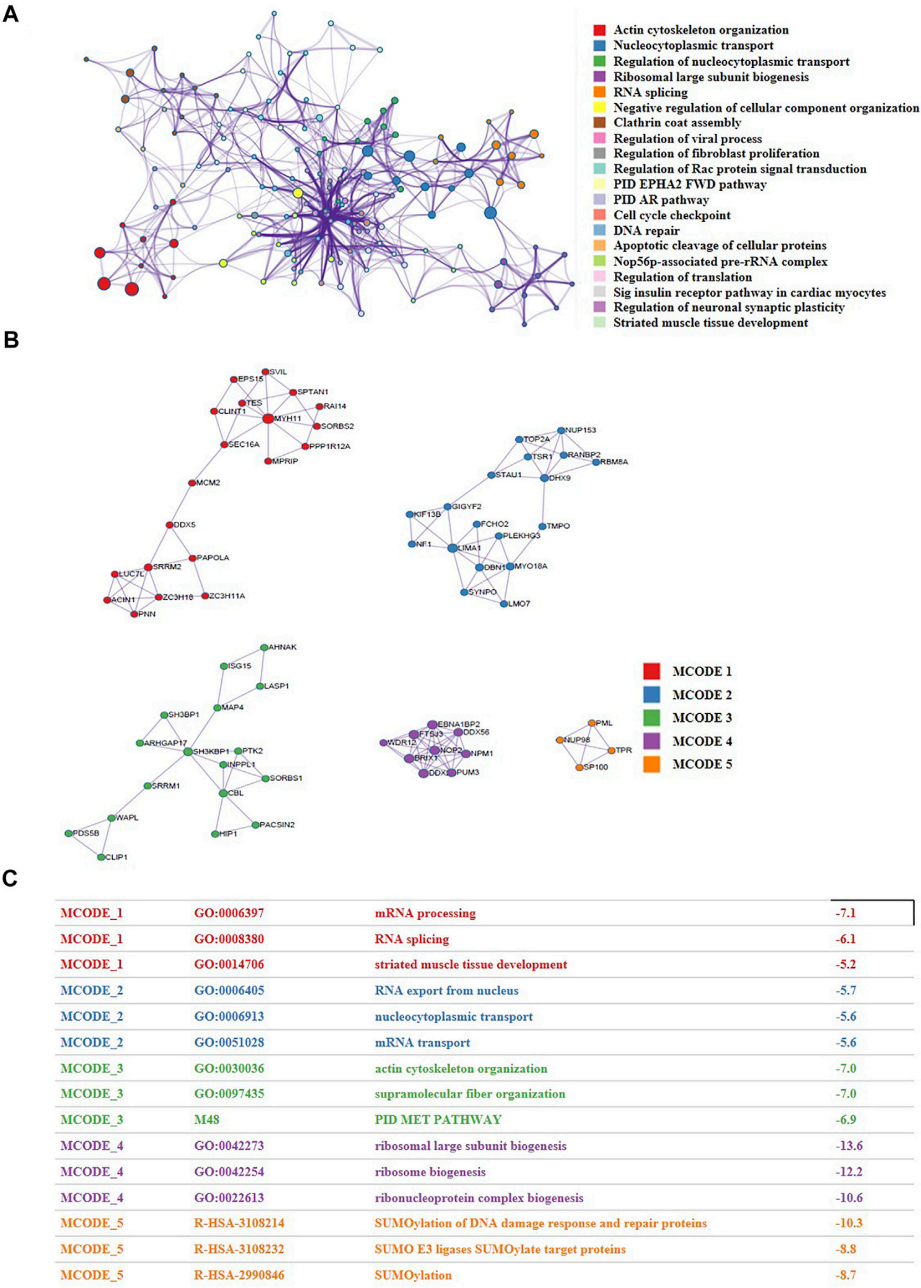
The proteomic and phosphoproteomic investigations uncovered the network of nuclear proteins in CRC patients and identified the functional modules within the network. **(A)** A functional network characterized by altered nuclear proteins and phosphorylation of CRC cells versus normal cells. **(B,C)** Five closely-tied groups within the network.

The alterations of content and phosphorylation may affect the activity of proteins. To explore the network of nuclear proteins, we performed PPI analysis and function enrichment analysis of all the differentially expressed proteins and phosphorylation using Metascape. As shown in Figure 4A, the biological functions of all the expression- or phosphorylation-altered proteins were mainly involved in actin cytoskeleton organization, nucleocytoplasmic transport, ribosomal large subunit biogenesis, RNA splicing, and regulation of cellular component organization. Besides, five closely-tied protein group were enriched, whose functions were relevant to mRNA processing, mRNA transport, PID MET pathway, and SUMOylation (Figures 4B,C). It has been reported that SUMOylation are closely related to cancer initiation and progression (Xie et al., 2020). There is a crosstalk between ubiquitination and SUMOylation in cells (Chelbi-Alix and Thibault, 2021), and SUMOylation plays a significant role in maintaining genomic stability via SUMO-targeted ubiquitin ligase (STUbL) (Nie and Boddy, 2016). Interestingly, we also found a significant change of ubiquitination pathway when we performed KEGG enrichment analysis of all down-regulated phosphorylation in CRC cells versus the normal cells (Supplementary Figure S2). Consequently, we conjectured that there may be a crosstalk between SUMOylation and ubiquitination in CRC cells as well, which synergistically promotes the development of CRC (Figure 3G).

The multi-platform-based investigation uncovered the TFs potentially regulating the activity of nucleus of CRC patients.

**FIGURE 5:**
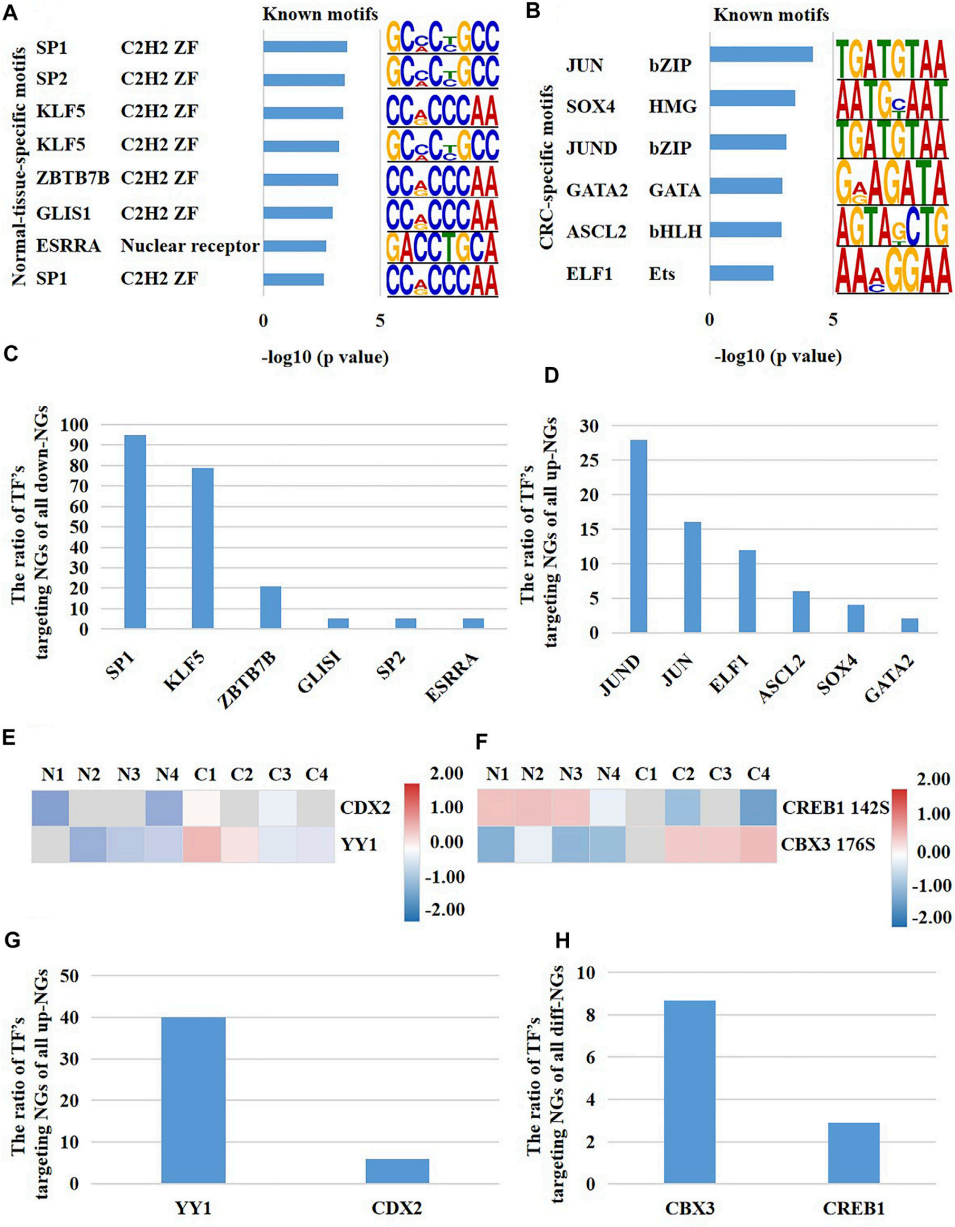
A multi-platform-based analysis disclosed the upstream TFs potentially regulating the activity of nucleus in CRC patients. **(A)** The normal-cell-specific TFs, and **(B)** the CRC-cell-specific TFs reflected by binding motifs. The proportion of TF’s potentially targeting nuclear genes of all **(C)** down-regulated nuclear genes and **(D)** up-regulated nuclear genes of CRC cells versus normal cells. Potential TFs which targeted the differentially expressed nuclear genes of CRC cells versus normal cells were altered on **(E)** the protein expression and **(F)** phosphorylation. The proportion of TF’s potentially targeting nuclear genes of **(G)** the up-regulated nuclear genes and **(H)** all differentially expressed nuclear genes. NGs represented nuclear genes.

The transcription of genes is regulated by their upstream TFs. In our above results, the mRNA expression of a large number of nuclear genes were altered in CRC cells versus normal cells, which prompted us to investigate whether their upstream TFs had abnormal molecular changes. We inquired all the nuclear genes which had different expression at both mRNA and protein levels through hTFtarget database. Subsequently, we obtained 144 experimentally confirmed TFs which were identified in colonic cells (Supplementary Table S1). TFs bind unique sequences (motif) of promoter to launch gene transcription. Consequently, we first analyzed the highly opened motifs in CRC cells and para-cancerous cells through comparing the global chromatin accessibility. The results showed that the binding motifs of TFs SP1, SP2, KLF5, ZBTB7B, GLIS1, and ESRRA were highly opened only in para-cancerous cells and potentially regulated 94.7, 78.9, 21.1, 5.3, 5.3, and 5.3% of all down-regulated nuclear genes, respectively (Figures 5A,C). On the other hand, the binding motifs of TFs JUN, SOX4, JUND, GATA2, ASCL2, and ELF1 were only opened in CRC cells and potentially regulated 28, 16, 12, 6, 4, and 2% of up-regulated nuclear genes in CRC cells, respectively (Figures 5B,D). Next, we evaluated the protein content and phosphorylation of the 144 TFs in CRC cells and normal cells. As a result, CDX2 and YY1 showed an increased protein expression in CRC cells versus normal cells (Figure 5E), and CDX2 and YY1 probably targeted 6 and 40% of all up-regulated differentially expressed nuclear genes, respectively (Figure 5G). Meanwhile, the phosphorylation of CBX3 at Ser176 and CRBE1 at Ser142 were down-regulated and up-regulated in CRC cells versus normal cells, respectively (Figure 5F), and CBX3 and CREB1 probably targeted 8.7 and 2.9% of all differentially expressed nuclear genes (Figure 5H). In summary, these results identified the potentially functional TFs of nuclear genes in CRC cells, including SP1, KLF5, JUND, JUN, ELF1, YY1, and CBX3.

The SVs of nuclear genes were potentially associated with the disorder of immune microenvironment in CRC patients.

**FIGURE 6:**
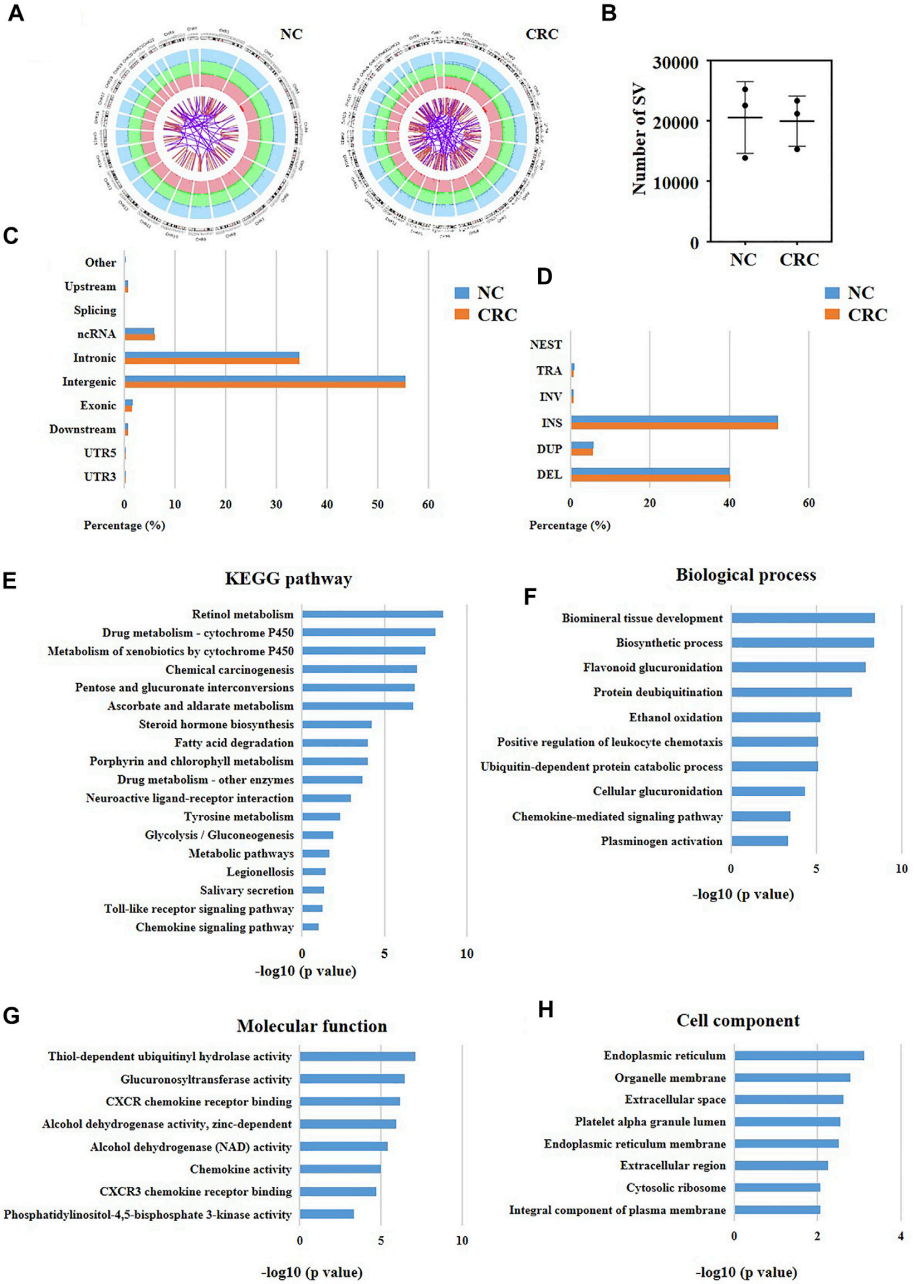
Identification of potential pathogenic SVs in CRC patients. **(A)** Circos plot showing the whole picture of SVs of normal cells and CRC cells. The outermost circle records different chromosomes. The second and third circles record different types of SVs. In the second circle, dark green represents insertion, dark blue represents deletion, and dark red represents amplification (light green, light blue, and light red are the corresponding background colors). In the third circle, red represents inversion, and purple represents interchromosomal translocations. NC represented normal cells. **(B)** The numbers of SVs of normal cells and CRC cells. **(C)** The located sites and **(D)** types of SVs of normal cells and CRC cells. **(E–H)** KEGG and GO analysis of the nuclear genes with CRC-specific SVs at exons.

SVs are a type of chromosome variation, characterized by large fragments (>50bp) of deletion, duplication, inversion, and translocation (Escaramís et al., 2015). Chromosome instability and specific SVs may cause tumors (Li et al., 2020). Consequently, we compared all SVs of CRC cells (Figure 6A) with SVs of normal adjacent cells using our original LWGS datasets (Figure 6B). Next, we calculated the numbers of SVs in CRC cells and normal cells, and found that there was no significant difference between these two groups (Figure 6C). Also, we analyzed the loci and types of SVs of CRC cells and normal cells, and the results showed that there was no significant difference between these two groups either (Figures 6D,E). We observed that the SVs located at introns and intergenes the most (Figure 6D), and insertion and deletion were the most common SVs types (Figure 6E). Since there was no significant difference in the overall number, type, and site of SVs between CRC cells and normal cells, we speculated that the specific SVs of CRC cells might be associated with the development of CRC. Hence, we screened out the CRC-specific SVs and obtained 1905 SVs ultimately (Supplementary Table S1). It was well known that exons contributed to protein translation in eukaryotes, which reminded us that SVs at exons might be associated with the initiation of CRC. Therefore, we classified CRC-specific SVs which located at exons into four categories. The SVs recorded in the Copy Number Variations and Related Diseases (CNVD) (a database recording malignant SVs) (Qiu et al., 2012) but not in the Genomic Variants database (DGV) (a database recording benign SVs) (MacDonald et al., 2014) were regarded as high-risk SVs. The SVs recorded in both CNVD and DGV databases were regarded as moderate-risk SVs. The SVs recorded only in DGV were regarded as low-risk SVs. The SVs with no records in any databases were regarded as possibly-deleterious SVs. Consequently, we identified one high-risk, nine moderate-risk, 29 probable-risk and 31 unknown-risk SVs in total (Supplementary Table S1).

To determine the functions of these high-risk and moderate-risk SVs, we further performed KEGG (Figure 6E) and GO (Figures 6F–H) analyses. The KEGG result showed that these SVs were mainly enriched in immune-related pathways, including toll-like receptor signaling pathway and chemokine signaling pathway (Figure 6E). Similarly, we also discovered that some immune pathways were enriched using GO analysis, such as positive regulation of leukocyte chemotaxis and chemokine-mediated signaling pathway. These results suggested that the CRC-specific SVs might be relevant to the dysfunctions of immune microenvironment in CRC patients.

**FIGURE 7:**
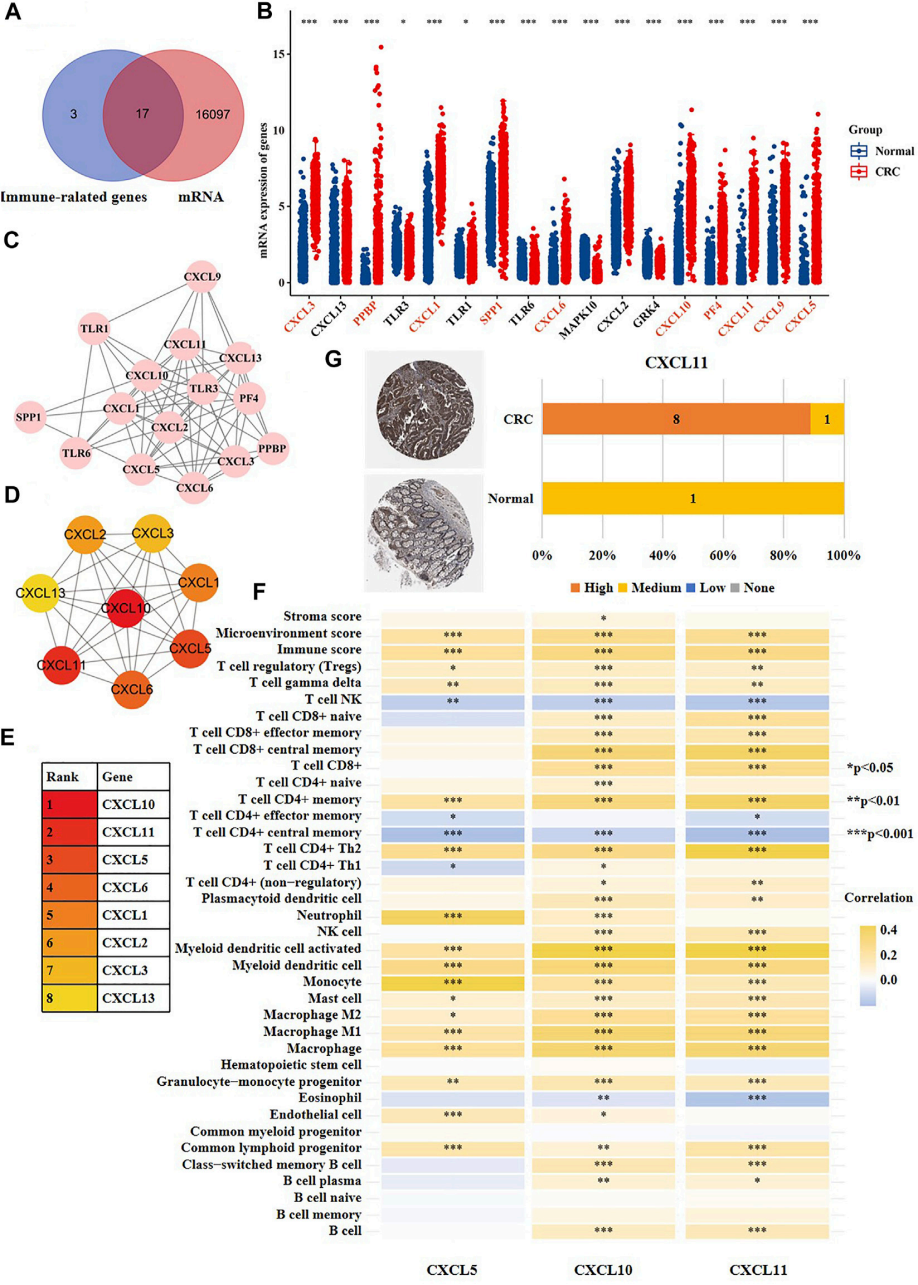
The CRC-specific SVs might affect immune microenvironment of CRC patients. **(A)** The overlapping analysis of 20 immune-related genes (purple) that developed SVs at exons and all differentially expressed genes of CRC cells versus normal cells (red). **(B)** The mRNA expression of 17 genes screened out from Figure 7A in CRC cells versus normal cells. The red color indicated that the labeled gene was up-regulated in CRC cells versus normal cells, and the black color indicated that the labeled gene was down-regulated in CRC cells versus normal cells. **(C)** The network of the 17 genes. **(D,E)** The top eight hub genes among the 17 genes. **(F)** The correlation between gene expression of CXCL5, CXCL10, CXCL11 and stroma score, microenvironment score, immune score and immune infiltration. **(G)** The immunohistochemical result of CXCL11 in human normal colon tissues and CRC tissues.

In recent years, immunotherapy has achieved great curative effects in CRC treatment [ (Becht et al., 2016; Kather et al., 2018; Ganesh et al., 2019)]. However, more reliable theoretical guidance is still needed for the selection of patents treated with immune drugs. In the above results, we observed that high-risk and moderate-risk SVs of CRC were highly enriched in immune-related pathways. Consequently, we further studied these immune-related genes which harbored CRC-specific SVs. Shown in Figures 7A,B, we found that 17 of the 20 immune-related genes had a changed mRNA expression based on the data sets from TCGA. We then investigated the network of the 17 immune-related genes using Cytoscape (Figure 7C). Subsequently, we searched for the hub genes within the network using Cytohubba. Shown in Figures 7D,E, the top eight genes were CXCL5, CXCL10, CXCL11, CXCL6, CXCL1, CXCL2, CXCL3, and CXCL13. We observed that these genes were all chemokine ligands. We further analyzed the association between the expression of the top three hub genes and immune microenvironment using the datasets from Xcell database. The results showed that CXCL5 expression was significantly correlated with microenvironment score, immune score, and the infiltration of regulatory T cells (tregs), gamma delta T cells, natural killer (NK) T cells, CD4^+^ memory T cells, T helper two cells (Th2), T helper one cells (Th1), neutrophils, and a total of 19 immune cells (Figure 7F). CXCL10 expression was associated with stroma score, microenvironment score, immune score, and the infiltration of tregs, T cell gamma delta, NK T cells, CD8^+^ naive T cells, CD8^+^ effector memory T cells, CD8^+^ central memory T cells, CD8^+^ T cells, CD4^+^ naive T cells, CD4^+^ memory T cells, and a total of 30 immune cells (Figure 7F). CXCL11 expression was associated with microenvironment score, immune score and the infiltration of tregs, gamma delta T cells, NK T cells, CD8^+^ naive T cells, CD8^+^ effector memory T cells, CD8^+^ central memory T cells, CD8^+^ T cells, CD4^+^ T cells, and a total of 27 immune cells (Figure 7F). Furthermore, we validated the protein expression of the highlighted genes CXCL11, CXCL10, and CXCL5 using the immunohistochemical results from the Human Protein Atlas, and found that the staining intensity of CXCL11 was higher expressed in CRC tissues versus normal colon tissues (Figure 7G), which was consistent with the mRNA result. However, CXCL5 was not detected in both normal colon tissues or cancer tissues, and there was no immunohistochemical results of CXCL10 in the database. The above results indicated that the SVs of CXCL5, CXCL10, and CXCL11 might be associated with the disturbance of immune microenvironment of CRC patients.

## Discussion

Fully understanding of the pathogenesis of cancer is helpful to screen therapeutic targets for clinic. As one of the most active organelles, the nucleus regulates genetic storage, replication, and transcription in eukaryotic cells. In our study, we examined the global SVs, DNA methylation, chromatin accessibility, proteome and phosphoproteome of CRC cells versus normal cells. As a result, we observed that several nuclear genes were altered at multiple molecular layers in CRC patients, such as UTP18, ANKS4B, KBTBD11, and PKP2. Frequently, genes with multiplex-level molecular changes may play a more important role in tumorigenesis. It is noteworthy that UTP18 has molecular changes at four levels, including protein expression, phosphorylation, DNA methylation, and chromatin accessibility. Studies have demonstrated that UTP18 is associated with preribosomal RNA splicing, ribosomal biogenesis, and translation maintaining. The over-expression of UTP18 in neuroblastoma promotes the expression of VEGF, Bcl-2, HIF1α, and c-MYC, improves the adaptability of tumor cells to environmental stress, and reduces the death of neuroblastoma cells after exposure to hydrogen peroxide, hypoxia or glucose deprivation (Yang et al., 2015). Additionally, a comprehensive bioinformatics study have also revealed that high expression of UTP18 promotes the growth of CRC cells (Hu et al., 2019).

Our study proclaimed an tandem and multi-layer crosstalk between phosphorylation and ubiquitination in CRC cells. It is well known that post-translational modification is crucial for regulating biological functions and dynamic activities of proteins. Our phosphoproteomic results showed that the phosphoric-acid-modified nuclear proteins involved in a series of ubiquitination-relevant processes, including DNA damage response and repair protein ubiquitination and E3 ligase target protein ubiquitination. Ubiquitination exists in almost all eukaryotic cells and is associated with the maintenance of genomic integrity, cell cycle, immune response, and intracellular signal transduction (Han et al., 2018). Meanwhile, we also observed a crosstalk between ubiquitination and SUMOylation in CRC cells. Many studies have demonstrated that the disorder of SUMOylation may lead to the development of diseases [ (Eifler and Vertegaal, 2015; Rabellino et al., 2017; Seeler and Dejean, 2017)]. Our study disclosed the interaction between phosphorylation, ubiquitination and SUMOylation, and highlighted their collaborative work in the development of CRC.

Furthermore, we revealed the TFs that potentially regulated the activity of nucleus in CRC cells, including SP1, SOX4, KLF5, JUND, JUN, YY1, CDX2, CBX3, CREB1. Among them, SP1 has been well studied in tumors [ (Vizcaíno et al., 2015; Bajpai and Nagaraju, 2017; Chen et al., 2018)]. Interestingly, the previous study has uncovered that SP1 mediates the bidirectional communication between mitochondria and nucleus. The functional changes of mitochondria can activate SP1, and then affect the transcription of a series of downstream genes (Soledad et al., 2019). This suggests an interaction between mitochondria and nucleus in CRC cells via SP1. YY1 is also a well-known oncogene. YY1 can maintain the survival of tumor cells and promote adaptation of cells to tumor microenvironment by regulating numerous genes related to cell proliferation, cell cycle, and inflammatory response [ (Meliala et al., 2020)].

Besides, we disclosed that the CRC-specific immune-relevant SVs were mainly enriched in toll-like receptor pathway and chemokine pathways. Many reports have demonstrated the toll-like receptor signaling is associated with the activation of both innate and adaptive immune responses, and has an core role in the initiation and development of CRC [ (Yang et al., 2017; Tintelnot and Stein, 2019; Rezasoltani et al., 2020)]. Currently, several drugs against toll-like receptor ligands (TRLs) have been approved by Food and Drug Administration (FDA) for cancer treatments, such as Bacillus calmette, monophosphoryl lipid A, and imiquimod [ (Moradi‐Marjaneh et al., 2018; Le Naour et al., 2020)]. Our results reveal new potential therapeutic targets of toll-like receptor pathway for CRC treatments.

The immunohistochemical results showed that CXCL5 was not detected in both normal colon tissues or cancer tissues, while its mRNA expression was up-regulated in CRC cells versus normal adjacent cells. As we know, gene expression is divided into two dimensions, including the mRNA level and protein level. According to a large number of studies, the protein abundance in cells is obviously different from mRNA in terms of half-life, synthesis rate and quantity [ (Schwanhäusser et al., 2011; Rodriguez et al., 2019)]. Besides, there is frequently complex post-transcription processing after transcription, such as non-coding RNA and mRNA modification.

In conclusion, we characterized the molecular changes of nucleus of CRC cells at distinct levels, and uncovered the potential hub genes and drug targets using a multi-omics approach. To our best knowledge, this is the first that the panoramic description of the multi-level molecular changes of nucleus of CRC cells is provided. The findings of our study may improve the understanding of pathogenesis of CRC and provide novel biomarkers and drug targets for clinic.

## Data Availability

The datasets presented in this study can be found in online repositories. The names of the repository/repositories and accession number(s) can be found below: http://www.proteomexchange.org/, PXD021314. http://www.proteomexchange.org/, PXD021318. https://www.ncbi.nlm.nih.gov/, PRJNA693028.
