# Supplementary material for: Multi-Platform-Based Analysis Characterizes Molecular Alterations of the Nucleus in Human Colorectal Cancer
Source: Front Cell Dev Biol. 2022 Feb 21;10:796703. doi: 10.3389/fcell.2022.796703 (PMC8899079; doi:10.3389/fcell.2022.796703)
Supplement: Supplementary file 2 [file Image1.PDF]

Supplementary Figures

Supplementary Figure S1

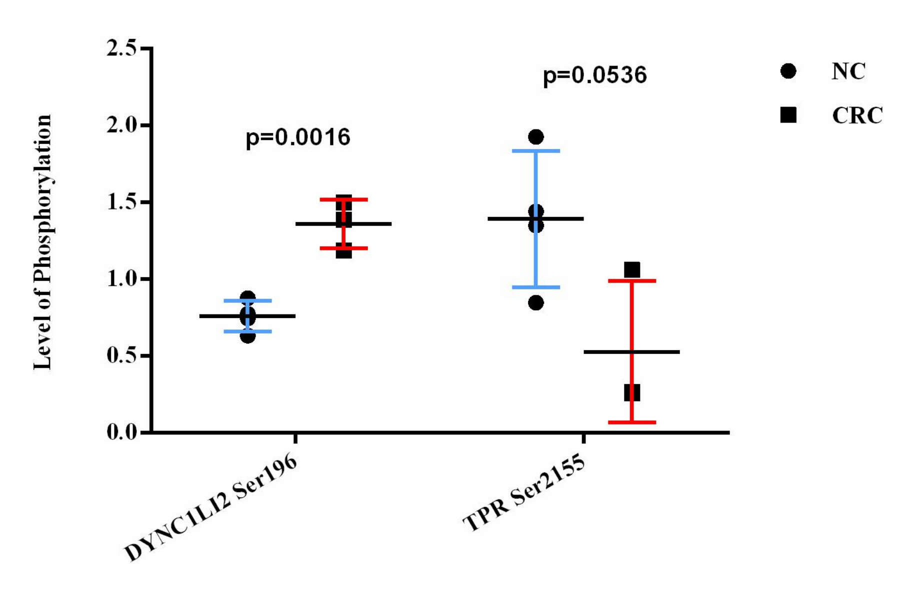

Supplementary Figure S1 The phosphorylation of DYNC1LI2 at Ser196 was up-regulated and TPR at Ser2155 was down-regulated in CRC cells versus normal cells.

Supplementary Figure S2

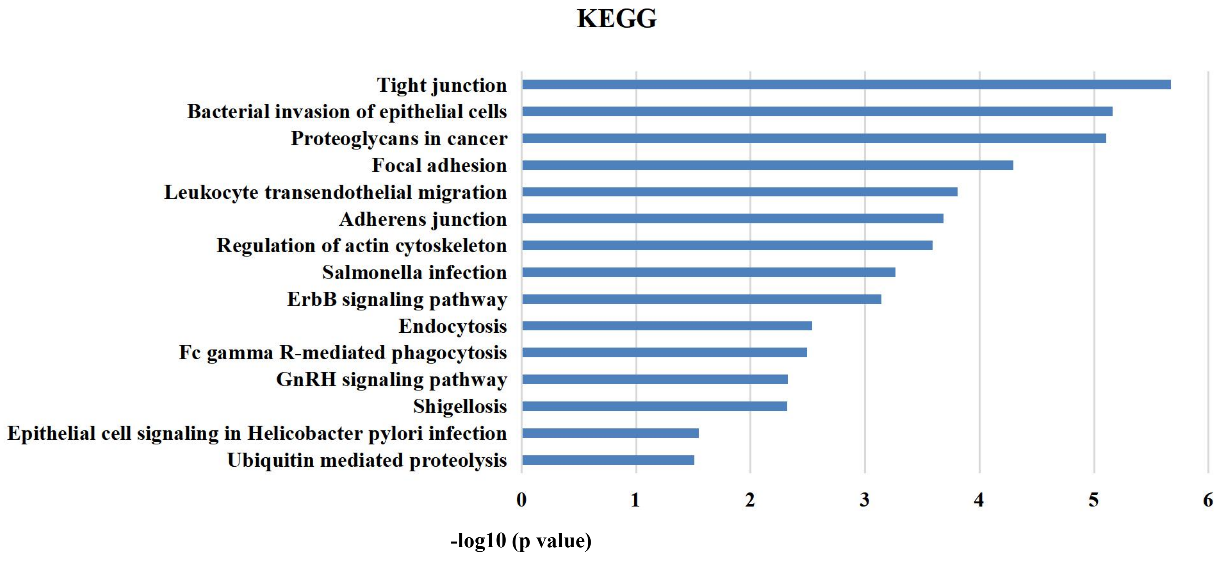

Supplementary Figure S2 KEGG analysis of all down-regulated phosphorylation of CRC cells versus normal cells.
